# The presence of heat-labile factors interfering with binding analysis of fibrinogen with ferritin in horse plasma

**DOI:** 10.1186/1751-0147-55-70

**Published:** 2013-09-22

**Authors:** Kazuma Takahashi, Takashi Kondo, Yasunaga Yoshikawa, Kiyotaka Watanabe, Koichi Orino

**Affiliations:** 1 Laboratory of Veterinary Biochemistry, School of Veterinary Medicine, Kitasato University, Aomori 034-8628, Japan; 2 Epizootic Research Center, Equine Research Institute, Japan Racing Association, 1400-4 Shiba, Shimotsuke-shi, Tochigi 329-0412, Japan

**Keywords:** Ferritin, Ferritin-binding protein, Fibrinogen, Heat-labile, Heme

## Abstract

**Background:**

Horse fibrinogen has been identified as a plasma specific ferritin-binding protein. There are two ways in the binding of ferritin-binding protein with ferritin: one is direct binding and the other is indirect binding which is heme-mediated. The aim of this study was to analyze the binding between horse fibrinogen and ferritin.

**Findings:**

Although fibrinogen in horse plasma did not show the binding to ferritin coated on the plate wells, after following heat-treatment (60°C, 30 min) of horse plasma, plasma fibrinogen as well as purified horse fibrinogen bound to plates coated with horse spleen ferritin, but not with its apoferritin which lost heme as well as iron after the treatment of reducing reagent. Binding of purified or plasma fibrinogen to ferritin was inhibited by hemin and Sn-protoporphyrin IX (Sn-PPIX), but not by PPIX or Zn-PPIX.

**Conclusions:**

Heat-treatment of horse plasma enabled plasma fibrinogen to bind to plate well coated with holo-ferritin. From the binding analysis of fibrinogen and ferritin, it is suggested that horse fibrinogen recognized iron or tin in complexed with the heme- or the hemin-ring, and also suggest that some fibrinogens circulate in the form of a complex with ferritin and/or heat-labile factors which inhibit the binding of fibrinogen with ferritin.

## Findings

Ferritin is a ubiquitous iron-binding protein found in animals, plants, and bacteria [1-3]. Mammalian ferritin stores iron atoms in the apoferritin shell that is composed of Heavy and Light subunits [1-3]. Intracellular ferritin also functions in storing a nontoxic form of iron to prevent its use in the production of hydroxyl radicals through the iron-mediated Fenton reaction [2-4]. Serum ferritin is present in relatively low concentrations (<1 μg/mL), and serum ferritin is the indicator of body iron stores [3]. Serum ferritin is also a biomarker of several inflammatory diseases as in human arthritis and bovine intramammary mastitis [3,5].

The following ferritin-binding proteins have been identified: H-kininogen [3,6], α_2_-macroglobulin [7,8], anti-ferritin autoantibodies [3], fibrinogen [9], apolipoprotein B [3], and α-casein [10]. With the exception of α-casein, these proteins most likely function in the removal of circulating ferritin [3,8]. Ferritin-binding proteins and ferritin may bind directly (e.g., binding between ferritin and H-kininogen or anti-ferritin autoantibody) [3,6] or indirectly (e.g., heme-mediated binding between ferritin and apolipoprotein B or α-casein) [3,11].

The purpose of this study was to determine the mechanism of horse plasma fibrinogen-ferritin binding. Horse plasma fibrinogen results in lower concentrations of ferritin in the plasma than in the serum, but heating at 75°C for 15 min results in equalization of both concentrations of ferritin [9,11], suggesting that fibrinogen binds circulating ferritin. However, in this study, the results of this binding analysis suggested the existence of heat-labile factors that inhibits binding between fibrinogen and ferritin.

Blood samples were collected from six thoroughbred horses (age 9–28 years) kept for the research at Kitasato University and the Equine Research Center, Japan Racing Association (Tochigi, Japan). Plasma was prepared from heparinized blood and was kept at 4°C in the presence of 0.1% sodium azide. All experiments were conducted by the following established guidelines for animal welfare and were approved by the Committee on the Ethics of Animal Experiments of the Kitasato University (Permit Number: 11–091).

Commercial horse spleen ferritin (Sigma Chemicals, St. Louis, MO, USA) was further purified [11] and its apoferritin was prepared by dialysis of holoferritin against 100 mmol/L thioglycolic acid in 100 mmol/L acetate buffer (pH 5.5). Horse plasma samples were diluted 100-fold with phosphate buffered saline (PBS; 150 mmol/L NaCl, 15 mmol/L dibasic and 5 mmol/L monosodium phosphate, pH 7.2) containing 0.1% Tween 20 and 0.1% gelatin (buffer A) and heated at 60°C for 30 min and then centrifuged at 14,000 × *g* for 15 min. The resulting supernatant was used as heat-treated plasma. Aliquots (100 μL) of holo- or apo-ferritin solutions in PBS (10 nmol/L each) were added to wells of a Maxisorp F96 immunoplate (Nunc, Roskilde, Denmark) and kept overnight at 4°C. The plate wells were washed three times with PBS containing 0.05% Tween 20 (PBST) after every step. After washing with PBST, 300 μL of buffer A was added to each well and masked with gelatin for 1 h to prevent nonspecific binding. To each well was also added 100 μL of horse fibrinogen (10 μg/mL) in ELISA buffer containing 10 mmol/L EDTA (buffer B) or the heat-treated plasma samples and the plate was incubated at 37°C for 2 h. After washing, 100 μL of goat anti-human fibrinogen antibody (GenWay Biotech, Inc., San Diego, CA, USA) diluted 1000-fold with buffer B was added to each well and the plate was incubated at 37°C for 1.5 h. The plate was then washed and 100 μL of alkaline phosphatase (ALP)-labeled rabbit anti-goat IgG antibody (EMD Merck Millipore, Billerica, MA, USA), which diluted 1000-fold with buffer A, was added to well and the plate was incubated at 37°C for 1.5 h. After washing, the enzyme reaction was performed using 3 mmol/L disodium *p-*nitrophenyl phosphate, and absorbance of each well at 405 nm was measured with Molecular Devices VersaMax™ Absorbance Tunable Microplate Reader.

Hemin, Sn-protoporphyrin IX (Sn-PPIX), Zn-PPIX, and metal-free PPIX were prepared as preciously described [10]. These inhibitors were simultaneously added to fibrinogen (10 μg/mL) in buffer B or heat-treated plasma (1:100 v/v) to a final concentration of 10 μmol/L. Aliquots (100 μL) of each mixture were added to holoferritin-coated wells (1 pmol/well) as described above. The detection of fibrinogen bound to the wells was performed as described above except for the use of buffer A in place of buffer B in every step. All data are expressed as the mean ± SD, and significant differences between data from the two groups was assessed using the Student’s *t*-test. The significant differences for multiple comparisons was assessed using one-way ANOVA followed by Tukey’s test. A *P*-value below 0.01 was considered statistically significant.

**Figure 1:**
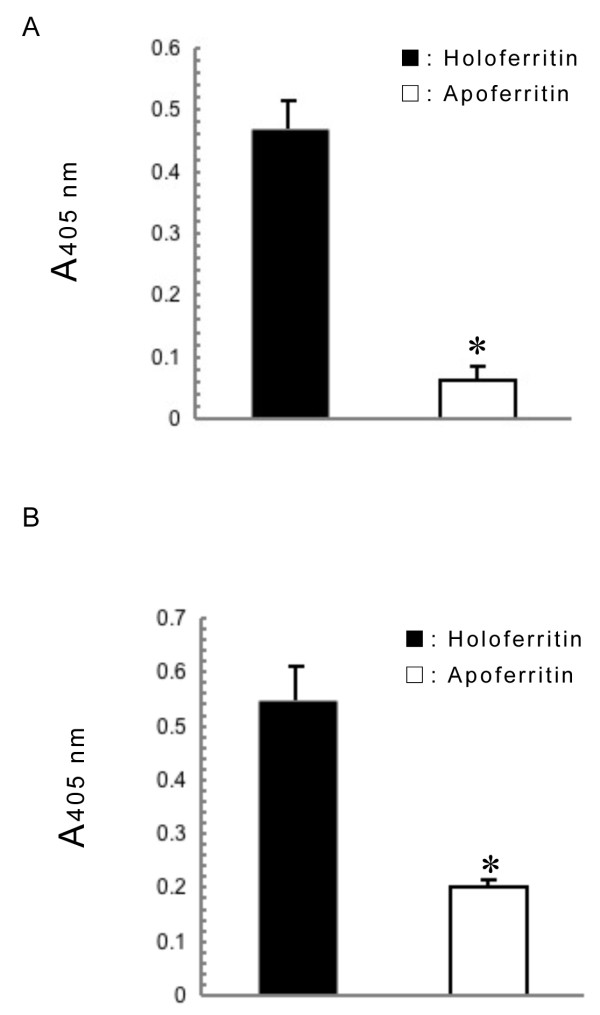
**Binding of horse fibrinogen and fibrinogen in horse plasma to holoferritin and apoferritin.** Plasma from 4 horses was diluted 100-fold with buffer A and heated at 60°C for 30 min, followed by centrifugation (14,000 × *g*, 15 min). Aliquots (100 μL) of purified horse fibrinogen (1 μg/well) **(A)** or resulting supernatant from heat-treated plasma **(B)** were added to wells of holo- or apo-ferritin-coated plate (1 pmol/well each). Fibrinogen bound to the wells was detected as using a goat anti-human fibrinogen antibody and an ALP-labeled rabbit anti-goat IgG antibody. Each data represents mean ± SD of four determinations or the average data from each horse. *: *P <* 0.01 compared to holoferritin.

**Figure 2:**
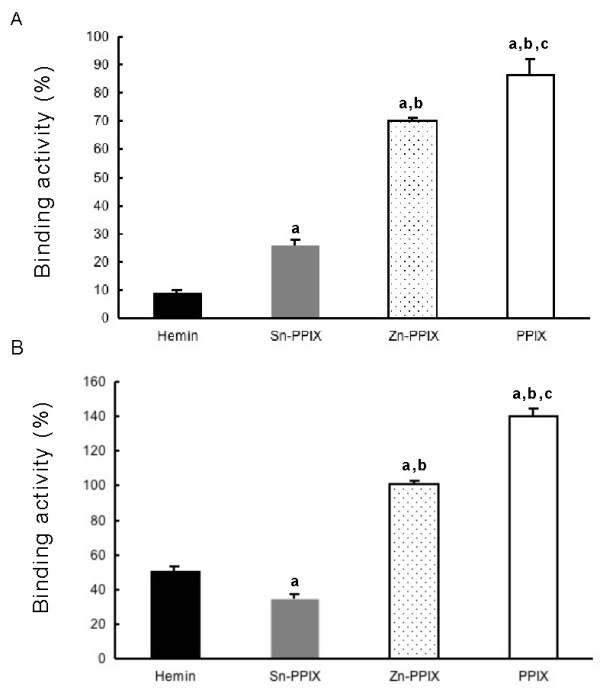
**Inhibition of the binding between holoferritin and purified fibrinogen or fibrinogen in heat-treated plasma by hemin, metal free-, Sn-, or Zn-PPIX.** Aliquots (100 μL) of 10 μg/mL of purified horse fibrinogen **(A)** or heated-horse plasma sample **(B)** as described in “Figure 1” were added to wells of holo-ferritin-coated plate (1 pmol/well) and hemin (solid bar), Sn- (gray bar), Zn- (dotted bar), or metal free-PPIX (open bar) was added to a final concentration of 10 μmol/L. Fibrinogen bound to the wells was detected as already described in “Figure 1”. Binding activity (%) was determined by comparison to the control (100%) in the absence of each inhibitor. a: *P <* 0.01 compared to the binding activity in the presence of hemin; b: *P <* 0.01 compared to the binding activity in the presence of Sn-PPIX; c: *P <* 0.01 compared to the binding activity in the presence of Zn-PPIX. Each data represents mean ± SD of four determinations.

The binding mechanism of plasma in horse plasma and ferritin has not been revealed due to non-binding of fibrinogen to ferritin. Subjecting horse plasma to heat treatment at 60°C for 30 min resulted in maximum binding of plasma fibrinogen to ferritin-coated microwells (Additional file 1: Data S1). Fibrinogen in heated-treated plasmas showed significant higher binding activity with holoferritin than with apoferritin as in purified horse fibrinogen (Figure 1A and B). Human fibrinogen showed heat stability (68°C, 10 h) in the pasteurization process [12]. However, heat denaturation of fibrinogen occurred by divalent cations such as Ca^2+^ (2 mmol/L) and Zn^2+^ (20 μmol/L) [13]. Therefore, a dilution (100-fold) of plasma may eliminate the possibility of fibrinogen denaturation by heat-treatment and divalent cations [14]. Therefore, we suggested that binding between fibrinogen and ferritin is heme-mediated as in apolipoprotein B and α-casein because reducing treatment of holoferritin releases heme as well as iron [3,10]. Binding between purified fibrinogen or plasma fibrinogen to holoferritin was significantly inhibited by hemin and Sn-PPIX, but not by Zn-PPIX or metal free-PPIX (Figure 2A and B) as calculated the binding activity (%) as 100% for the control in the absence of each inhibitor. This result agrees with the observation that Sn-PPIX is the most potent competitive inhibitor of heme oxygenase reacting with heme as substrate [15]. Binding between purified fibrinogen or plasma fibrinogen to holoferritin was not blocked by ferrous ammonium sulfate (Fe^2+^) or ammonium iron sulfate (Fe^3+^), even at a concentration of 1 mmol/L (data not shown), suggesting that the binding of fibrinogen to ferritin is not iron-dependent or not necessary for only iron. These results demonstrated that horse fibrinogen strongly recognizes iron or tin complexed with the heme- or the hemin-ring. Whereas the potency of the inhibitors used to block binding between purified fibrinogen and holoferritin was in the order hemin > Sn-PPIX > Zn-PPIX, Sn-PPIX was the most potent inhibitor when using heat-treated plasma, and Zn-PPIX did not show any inhibition. Additionally, PPIX enhanced the binding between plasma fibrinogen and holoferritin different from purified fibrinogen. The different inhibitory effects of various PPIX derivatives on the ferritin-binding in purified fibrinogen and plasma fibrinogen remains to be elucidated. Plasma contains factors such as hemopexin as a heme-binding protein [16] and albumin and α_2_-macroglobulin that bind Zn ion [17], suggesting that these interactions result in apparent lower inhibitor concentrations compared to purified fibrinogen. Plasma may contain some factors that interact with PPIX to enhance binding activity between fibrinogen and ferritin.

Why fibrinogen in untreated plasma did not bind ferritin-coated wells also remains to be determined. Watanabe et al. [18] reported that the structure of adenylate kinase changes due to denaturation as coated on plates, resulting in the antigenicity change. This finding led to the hypothesis that coating plasma proteins on the wells may release heat-labile factors which inhibit the binding of fibrinogen to coated ferritin. The fibrinogen concentration in this study (10 μg/mL) was estimated to be physiological concentration from plasma dilution (100-fold) and the normal range (1–2 mg/mL) [19]. This interference of heat-unstable proteins was observed even after at least one year storage of plasma sample at 4°C (data not shown), suggesting that plasma storage did not affect interaction between fibrinogen and them. It is known that fibrinogen binds IgG [20,21]. We detected binding between immunoglobulins in untreated plasma and fibrinogen trapped on the plate by coated anti-fibrinogen antibody (Additional file 2: Data S2). Horse IgGT and IgGb showed higher binding activity with fibrinogen. However, although an attempt to detect complexes formed between ferritin, fibrinogen, and immunoglobulin as fibrinogen-binding protein was not successful, this findings suggest that fibrinogen partly circulate as a complex with ferritin and/or a heat-labile fibrinogen-binding protein, probably IgG.

The high redox potential of iron and heme cause oxidative damage [3,22]. Ferritin and/or fibrinogen in horse circulation may protect iron- or heme-mediated oxidative stress. Additionally, the binding between them is likely to be involved in local blood coagulation as in H-kininogen [6]. This study may also provide preliminary data on physiological relation between iron metabolism and blood coagulation system.

In conclusion, plasma fibrinogen bound to coated ferritin only after plasma was heated (60°C, 30 min). Horse fibrinogen bound holo-ferritin, but not apo-ferritin also having no heme. Binding of fibrinogen to ferritin was inhibited by hemin and Sn-PPIX, but not by metal-free PPIX or Zn-PPIX. This study demonstrates that horse fibrinogen binds ferritin through heme-mediation, and that plasma heat-labile factors inhibit the binding between them.

## Competing interests

The authors declare that they have no competing interests.

## Authors’ contributions

Conceived ad designed the experiments: KT, TK, YY, KW, KO. Performed experiments: KT, KO. Analyzed data: KT, TK, KW, KO. Contributed reagents/ materials/ analysis tools: YY, KW, KO. Wrote the paper: KT. All authors read and approved the final manuscript.

## Supplementary Material

Additional file 1 **Data S1.** The effect of heat treatment of horse plasma on the binding of plasma fibrinogen to coated ferritin. Plasma from 3 horses was diluted 100-fold with buffer A was heated at the temperature indicated for 30 min, followed by centrifugation at 14,000 × *g* for 15 min. Aliquots (100 μL) of the resulting supernatant were added to wells of a commercial horse spleen ferritin-coated immunoassay plate (1 pmol/well). Fibrinogen bound to the wells was detected using a goat anti-human fibrinogen antibody and an ALP-labeled rabbit anti-goat IgG antibody. Data represents mean ± SD of the average data from each horse. Click here for file

Additional file 2 **Data S2.** The detection of immunoglobulin G binding to fibrinogen in plasma from 3 horses. Aliquots (100 μL) of sheep anti-human fibrinogen antibody (AbD Serotec, Inc., Raleigh, NC, USA) diluted with PBS were added to wells (90 μg/well), and the plate was kept overnight at 4°C. After washing and masking with gelatin, 100 μL of horse plasma diluted 200-fold with buffer A was added to the well, and incubated at 37°C for 2 h. After washing, 100 μL of monoclonal antibodies to IgGa, IgGb, IgG or IgGT diluted with buffer A was added to the wells. The immunoglobulin bound to fibrinogen in horse plasma was detected with an ALP-labeled goat anti-mouse IgG antibody (SouthernBiotech Assoc., Birmingham, AL, USA). Data represents mean ± SD of the average data from each horse. Click here for file
