# Supplementary figures and images for: The presence of heat-labile factors interfering with binding analysis of fibrinogen with ferritin in horse plasma
Source: Acta Vet Scand. 2013 Sep 22;55(1):70. doi: 10.1186/1751-0147-55-70 (PMC4016576; doi:10.1186/1751-0147-55-70)

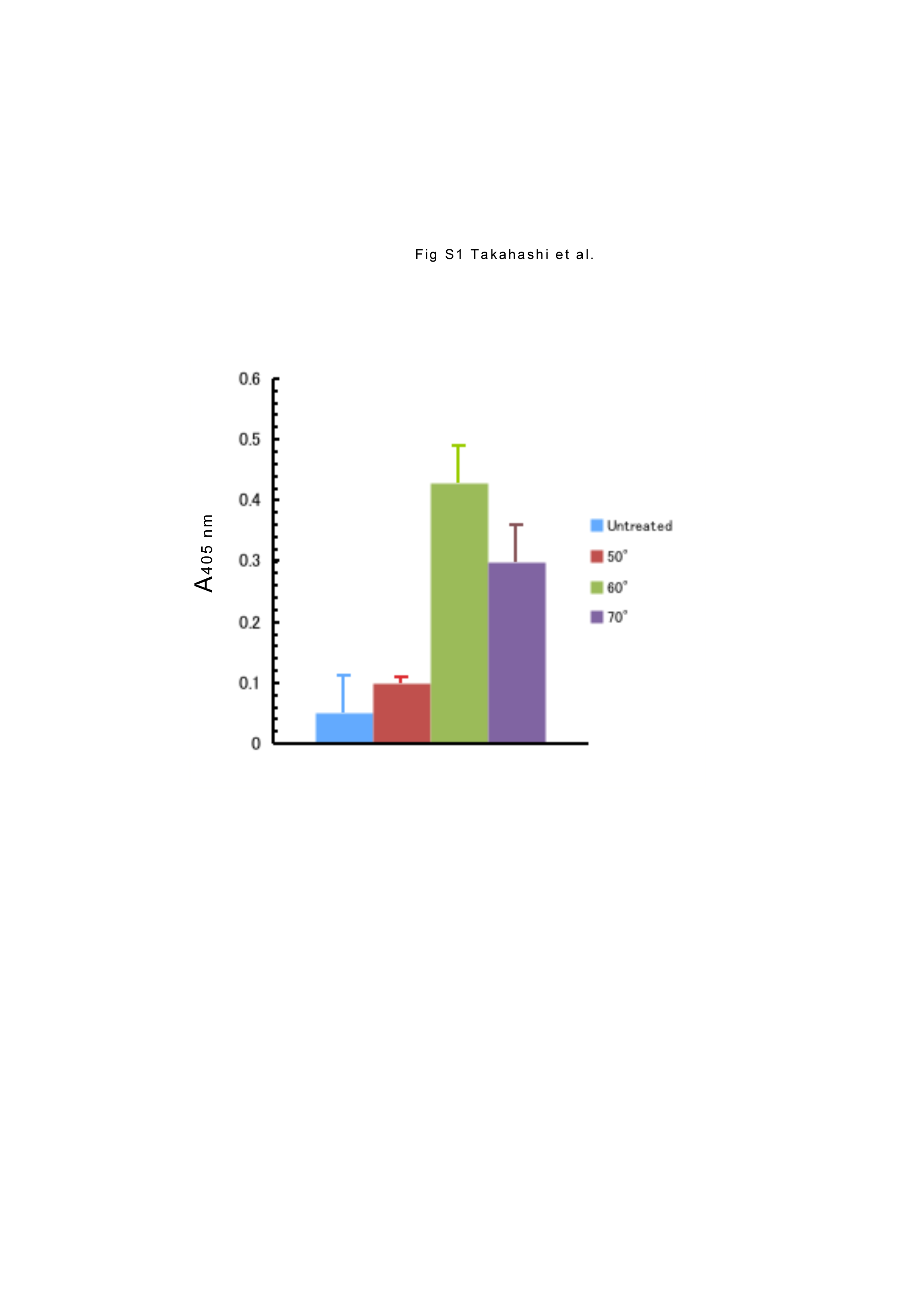

Supplement: Additional file 1 — Data S1. The effect of heat treatment of horse plasma on the binding of plasma fibrinogen to coated ferritin. Plasma from 3 horses was diluted 100-fold with buffer A was heated at the temperature indicated for 30 min, followed by centrifugation at 14,000 × g for 15 min. Aliquots (100 μL) of the resulting supernatant were added to wells of a commercial horse spleen ferritin-coated immunoassay plate (1 pmol/well). Fibrinogen bound to the wells was detected using a goat anti-human fibrinogen antibody and an ALP-labeled rabbit anti-goat IgG antibody. Data represents mean ± SD of the average data from each horse. [file 1751-0147-55-70-S1.tiff]

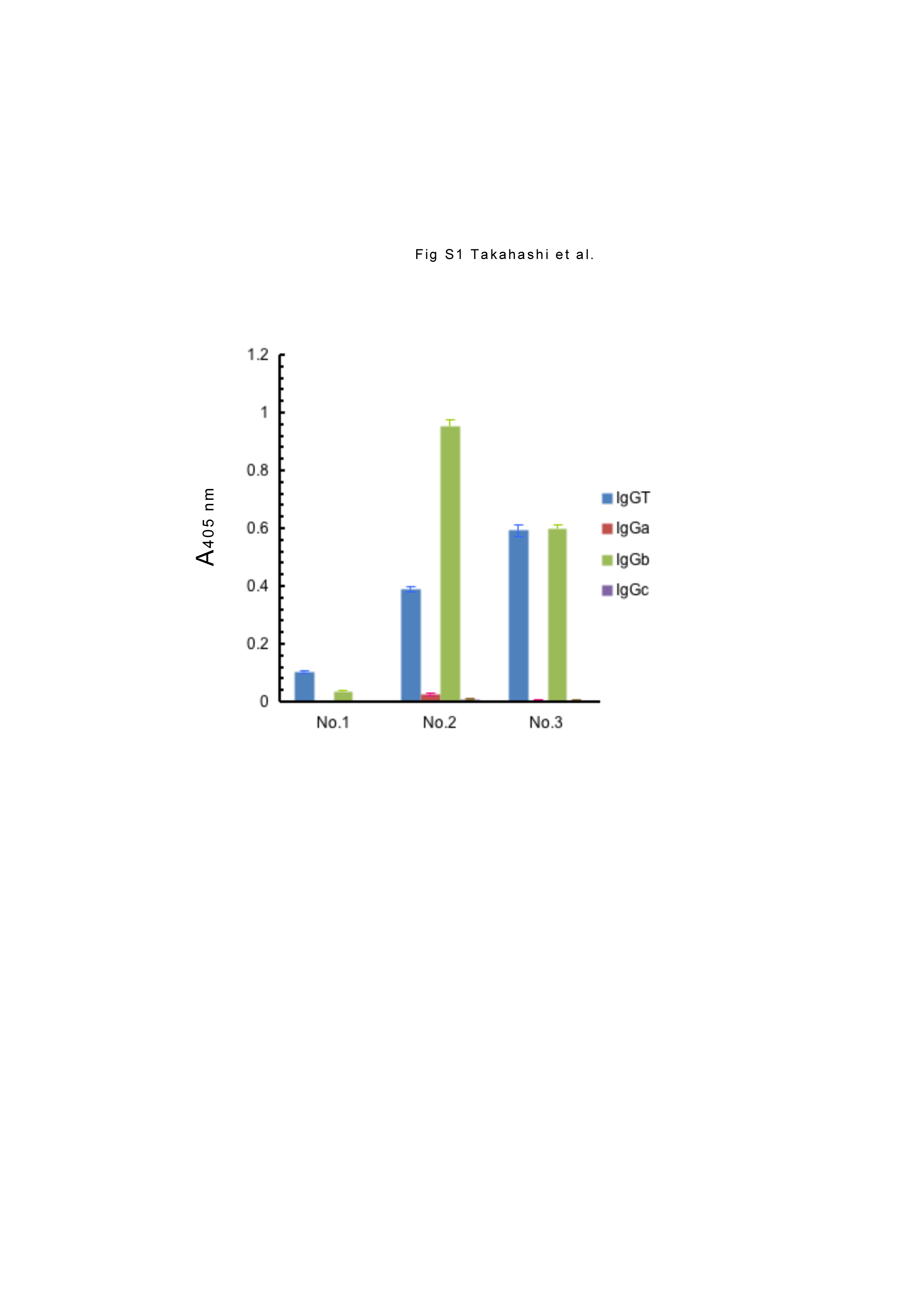

Supplement: Additional file 2 — Data S2. The detection of immunoglobulin G binding to fibrinogen in plasma from 3 horses. Aliquots (100 μL) of sheep anti-human fibrinogen antibody (AbD Serotec, Inc., Raleigh, NC, USA) diluted with PBS were added to wells (90 μg/well), and the plate was kept overnight at 4°C. After washing and masking with gelatin, 100 μL of horse plasma diluted 200-fold with buffer A was added to the well, and incubated at 37°C for 2 h. After washing, 100 μL of monoclonal antibodies to IgGa, IgGb, IgG or IgGT diluted with buffer A was added to the wells. The immunoglobulin bound to fibrinogen in horse plasma was detected with an ALP-labeled goat anti-mouse IgG antibody (SouthernBiotech Assoc., Birmingham, AL, USA). Data represents mean ± SD of the average data from each horse. [file 1751-0147-55-70-S2.tiff]
